# Medical News

**Published:** 1899-10

**Authors:** 


### SCOTT & BOWNE.

THIS firm did the handsome thing by reserving seats for visiting doctors in their stand, to witness the Dewey parade. We regret that ATLANTA JOURNAL-RECORD OF MEDICINE could not be present in place reserved for its occupation.

---

WE regret to announce the death, last month, of Mr. Chas. W. Bittmann, of St. Louis. He was our representative in some business there, and especially trustworthy and valued.

